# Association Between Level of Fecal Calprotectin and Progression of Crohn's Disease

**DOI:** 10.1016/j.cgh.2019.02.017

**Published:** 2019-10

**Authors:** Nicholas A. Kennedy, Gareth-Rhys Jones, Nikolas Plevris, Rebecca Patenden, Ian D. Arnott, Charlie W. Lees

**Affiliations:** ∗ Inflammatory Bowel Disease Pharmacogenetics, University of Exeter, Exeter, United Kingdom; ‡ Gastrointestinal Unit, Western General Hospital, Edinburgh, United Kingdom; ‖ Department of Clinical Chemistry, Western General Hospital, Edinburgh, United Kingdom; § Gastrointestinal Unit, University of Edinburgh, Edinburgh, United Kingdom

**Keywords:** IBD, Biomarker, Prognostic Factor, Noninvasive, CD, Crohn’s disease, FC, fecal calprotectin, IBD, inflammatory bowel disease, IQR, interquartile range

## Abstract

**Background & Aims:**

Mucosal healing is associated with improved outcomes in patients with Crohn’s disease (CD), but assessment typically requires ileocolonoscopy. Calprotectin can be measured in fecal samples to determine luminal disease activity in place of endoscopy—this measurement is an important component of the treat-to-target strategy. We investigated whether levels of fecal calprotectin are associated with subsequent CD progression.

**Methods:**

We performed a retrospective study of 918 patients with CD (4218 patient-years of follow-up evaluation; median, 50.6 mo; interquartile range [IQR], 32.8–76.0 mo) managed at a tertiary medical center in Edinburgh, United Kingdom, from 2003 through 2015. Patients were included if they had 1 or more fecal calprotectin measurements made 3 months or more after their diagnosis. We collected clinical data and fecal calprotectin measurements and analyzed these data to identify factors associated with a composite outcome of progression in Montreal behavior, hospitalization, and resection.

**Results:**

An increased level of fecal calprotectin at the index visit was associated with subsequent progression of CD, independent of symptoms or disease location. The median level of fecal calprotectin at the index visit was 432 μg/g (IQR, 1365–998 μg/g) in patients who reached the composite end point vs 180 μg/g (IQR, 50–665 μg/g) in patients who did not. In multivariable analysis, a cut-off value of 115 μg/g calprotectin identified patients who met the end point with a hazard ratio of 2.4 (95% CI, 1.8–3.1; *P* < .0001).

**Conclusions:**

In a retrospective analysis of patients with CD, we found that measurements of fecal calprotectin made during routine monitoring can identify patients at risk for disease progression, independent of symptoms or disease location. It is therefore important to screen asymptomatic patients for mucosal inflammation and pursue complete resolution of inflammation.

**What You Need to Know**

**Background**

Fecal calprotectin is a marker of luminal Crohn’s disease activity. We investigated whether fecal calprotectin is associated with subsequent Crohn’s disease progression.

**Findings**

We have shown that an increased fecal calprotectin level is associated with a long-term increase in disease progression, including hospitalization, surgery, and advances in Montreal behavior.

**Implications for patient care**

It is important to screen asymptomatic patients for mucosal inflammation and pursue complete resolution of inflammation.

Crohn’s disease (CD), a form of inflammatory bowel disease (IBD), is characterized by relapsing episodes of intestinal inflammation and the accumulation of irreversible digestive damage. Prognosis is highly variable between individuals,^1^ such that the identification of patients at greatest risk of poor outcomes is an urgent research priority. Some clinical phenotypes, such as disease location and environmental factors such as smoking, have been associated clearly with poorer outcomes.^2^, ^3^ However, accurate prediction remains difficult. Over the past decade, there has been a paradigm shift away from treating until symptom resolution and toward mucosal healing because persistent subclinical bowel inflammation leads to poorer outcomes.^4^, ^5^, ^6^, ^7^, ^8^ However, this typically has required ileocolonoscopy, which is invasive, expensive, and carries risk for patients.^9^

Fecal calprotectin (FC) has become well established as a biomarker of intestinal inflammation. Calprotectin is a 36.5-kilodalton protein that constitutes 60% of the contents of granules in neutrophils.^10^ Its use as a screening test to distinguish IBD from irritable bowel syndrome is well supported by multiple studies, with an area under the receiver operating characteristic curve of 0.95 in meta-analysis.^11^ Several groups have shown that FC correlates well with endoscopic measures of disease activity.^12^, ^13^, ^14^, ^15^, ^16^ There has been greater uncertainty of its role in small-bowel CD, however, more recently, FC has been shown to correlate well with both magnetic resonance imaging^17^ and capsule endoscopy findings.^18^, ^19^

The use of FC as a prognostic marker has been shown in the context of medically and surgically induced remission.^20^, ^21^, ^22^ In both contexts, baseline FC predicts disease flare over a follow-up period of 2 years, although there is also a notable increase in FC at 3 to 4 months before clinical disease flare. The recent CALM study has shown the effectiveness of a treat-to-target strategy incorporating FC in Crohn’s disease.^23^ However, it still has not yet been shown whether increases in FC, irrespective of clinical symptoms, are associated with disease progression. This information would provide further support to the principle of treating beyond symptoms.

We aimed to use a large, extensively phenotyped cohort of CD patients followed up over time to determine the value of FC to predict disease progression. We focused on end points associated with digestive damage: progression of Montreal behavior,^24^ surgical resection, or hospitalization for severe flare.

## Methods

This was a retrospective cohort study of CD patients managed at the Western General Hospital (Edinburgh, UK), a teaching hospital that cares for secondary- and tertiary-referred patients with IBD. The primary inclusion criteria were a diagnosis of CD and at least 1 FC level measurement more than 3 months after diagnosis. The a priori primary end point was a composite of progression in Montreal luminal disease behavior (B1 to B2/B3 or B2 to B3), hospitalization for flare, and resection surgery. These individual components also were defined as separate secondary end points. To reduce the possibility of merely measuring the FC level at the time of the disease flare that caused the end point, any events that happened within 90 days after the index FC were regarded as having already happened and were not included in the end point analysis.

We obtained FC data from the Edinburgh FC Registry, a record of every FC measurement performed in Edinburgh since its introduction in 2003. Patients in this initial cohort had their first FC measurement between 2003 and 2014, and were followed up until 2015. Fecal calprotectin measurements were requested as part of routine monitoring and also as directed by patient symptoms. These data represent a convenience sample, and include all patients tested during that period who met our inclusion criteria.

We matched these data to existing research and clinical databases to identify patients with a known diagnosis of CD. We then searched the electronic and paper medical records to obtain information on demographics, symptoms, disease location, and behavior over time, hospitalizations, surgical procedures, investigations, and drug therapy. Disease location and behavior were classified according to the Montreal classification.^24^ Changes in disease behavior were defined as occurring when the first investigation that showed the change was performed, for example, a magnetic resonance imaging scan showing stricturing small-bowel disease.

Patients were regarded as symptomatic either by a Harvey Bradshaw Index measure greater than 4 and/or by physician global assessment of active symptomatic luminal disease.^25^ Each of the previous medical therapies was categorized as having ever taken vs never, with immunomodulators defined as azathioprine, mercaptopurine, and methotrexate. Data were stored in a Microsoft Access 2003 database (Microsoft, Redmond, WA).

FC collection kits were given to patients and samples were returned to the hospital biochemistry laboratories either directly or via their general practioner (samples forwarded the same day). Upon arrival at the laboratories, samples are stored at -20°C. FC was measured using a standard enzyme-linked immunosorbent assay technique (Calpro AS, Lysaker, Norway). All assays were performed using the same protocol in the Department of Clinical Biochemistry at the Western General Hospital (Edinburgh, UK). The manufacturer’s reference range for distinguishing IBD from functional gut disorders is higher than 50 μg/g.

Statistical analysis was performed using R 3.5.1 (R Foundation for Statistical Computing, Vienna, Austria). The Mann–Whitney *U* test was performed for continuous nonparametric data, and the Fisher exact test was used for categoric data. Survival analysis was performed using Kaplan–Meier and Cox proportional hazards models.^26^ For the survival models, we have reported the outcome as the proportion with maintained digestive health (ie, the inverse of our primary end point). Patients were excluded from the specific analysis of progression in Montreal behavior if they already were B3 at baseline.

FC was analyzed using log-transformed data and using a predefined threshold of 250 μg/g. The optimum threshold for FC on survival analysis then was explored by examining the *P* values of the likelihood ratio test and the Akaike Information Criteria for Cox proportional hazard models. Variable selection for multivariable models was performed using a stepwise backward method based on Akaike Information Criterion. We performed Cox proportional hazards analyses of the effect of drug therapy up to 3 months before or 6 months after fecal calprotectin on the primary outcome; for this analysis, patients who had disease progression within the first 6 months or who were censored in that period were excluded from analysis. The multistate transition data for disease progression in the overall cohort was performed using the empiric transition matrix method.^27^

The principal analysis was performed using the first FC measurement for each patient for those in whom there was more than 1. Because of the retrospective nature of this data set, these were not taken at uniform intervals. Exploratory analysis of multiple FC measurements was performed using the median for each rolling 6-month period centered on each month after diagnosis and stratified by progression in Montreal behavior. FC measurements were excluded from this analysis if the patient was symptomatic at the time of sampling.

This study was conducted as a service evaluation using data collected routinely as part of clinical care, and therefore following guidance from the UK Health Research Authority did not require specific ethical approval or consent.

## Results

**Figure 1:**
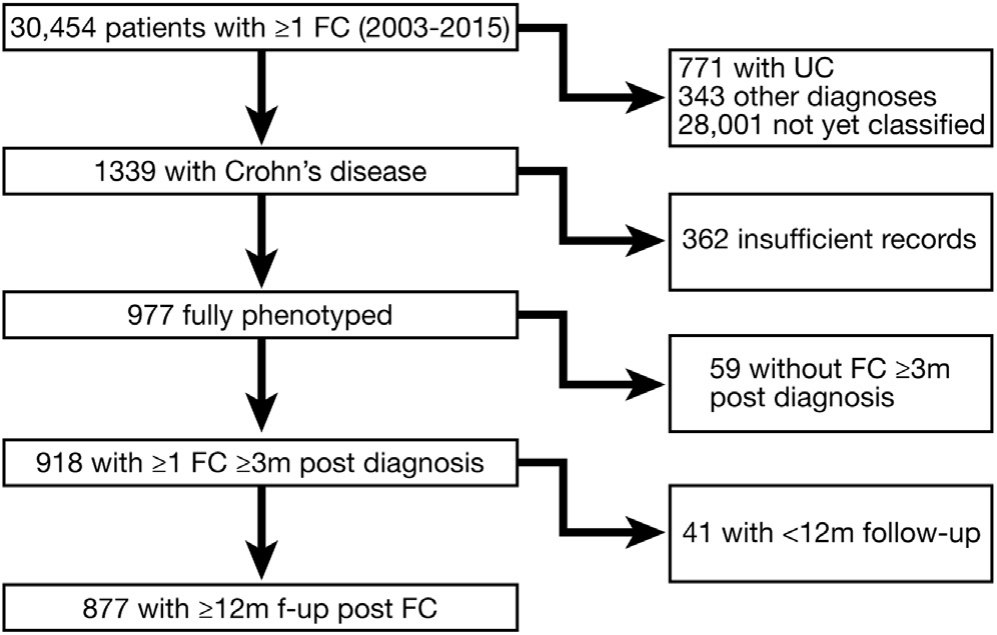
Derivation of the cohort of patients with Crohn’s disease, fecal calprotectin (FC), and follow-up data. UC, ulcerative colitis.

**Figure 2:**
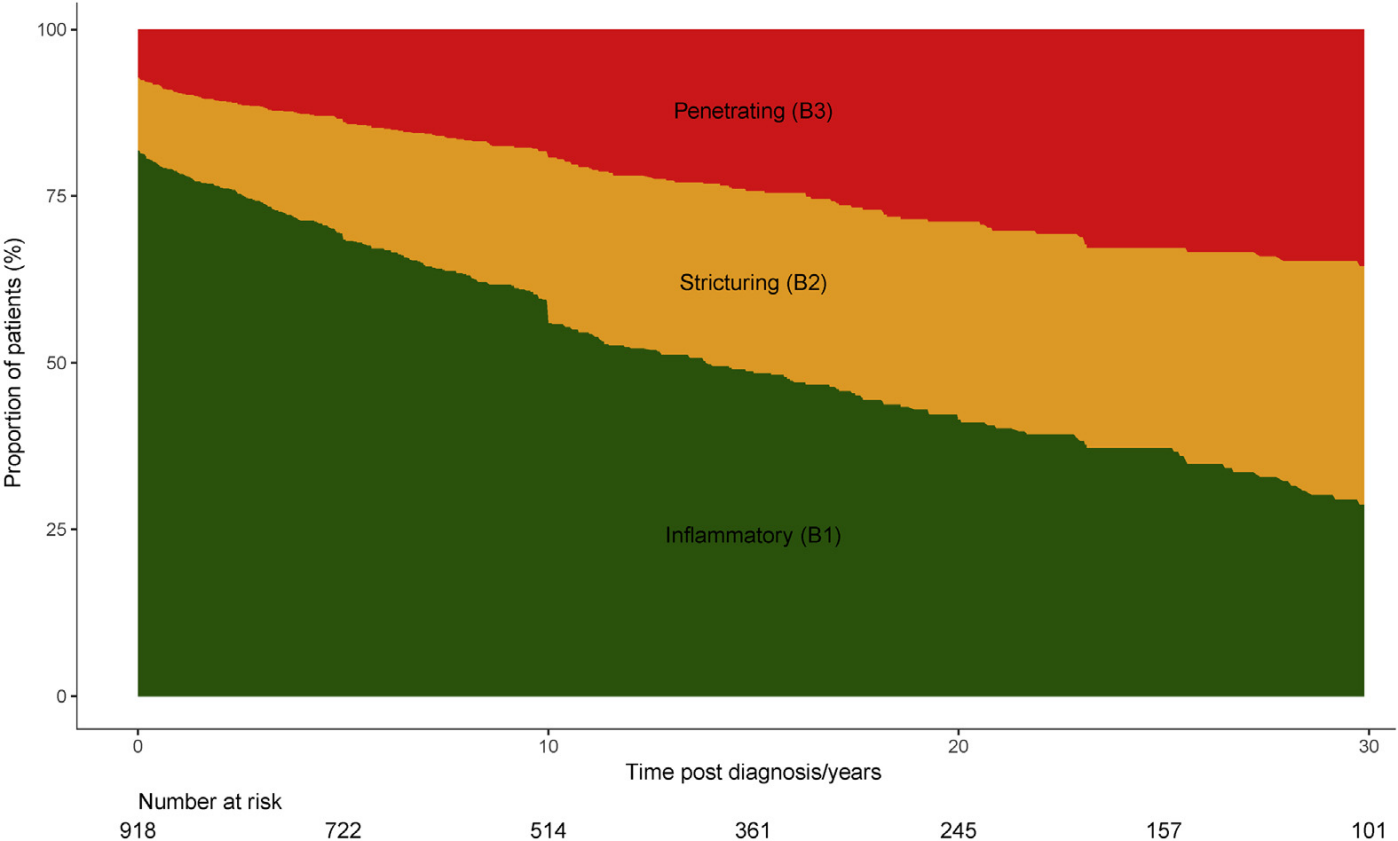
Disease progression over time in the whole cohort as estimated by the empiric transition matrix method.

We identified 918 CD patients meeting our inclusion criteria (Figure 1). A total of 61.1% were female, and the median age at the index FC measurement was 40.7 years (interquartile range [IQR], 28.5–54.8 y) (Table 1). The median follow-up time was 50.6 months (IQR, 32.8–76.0 mo), with a total of 4218 patient-years of follow-up evaluation across the cohort. At diagnosis, 81% had an inflammatory (B1) phenotype, 12% had a stricturing (B2) phenotype, and 8% had a penetrating (B3) phenotype. By 30 years after diagnosis, the proportions of B1, B2, and B3 phenotypes were estimated to be 29%, 36%, and 36%, respectively (Figure 2). The FC measurement was significantly higher in patients with L3 (median, 315; IQR, 90–866 μg/g) and L2 disease (median, 289; IQR, 69–909 μg/g) than in those with L1 disease (median, 180; IQR, 65–445 μg/g; *P* < .0001).

**Table 1:** Baseline Demographics of the Cohort (n = 918)

Variable	Median (IQR)/number (%)
Sex	
Female	561 (61.1%)
Age at diagnosis, *y*	27.4 (20.1–42.8)
Age at calprotectin, *y*	40.7 (28.5–54.8)
Months to first calprotectin	75.5 (15.5–183.8)
Year of first calprotectin	2010 (2008–2011) (range, 2003–2014)
Smoking at diagnosis	
Current	229 (32.5%)
Ex-smoker	101 (14.3%)
Never	375 (53.2%)
Montreal location	
L1 ± L4	289 (31.7%)
L2 ± L4	328 (36.0%)
L3 ± L4	288 (31.6%)
Isolated L4	6 (0.7%)
Montreal behavior at diagnosis	
B1	741 (80.7%)
B2	106 (11.5%)
B3	71 (7.7%)
Montreal behavior at index calprotectin	
B1	564 (61.4%)
B2	200 (21.8%)
B3	154 (16.8%)
New medication in 3 months before FC	
Steroids	91 (9.91%)
Immunomodulator	58 (6.32%)
Anti-TNF	16 (1.74%)
Any of these	146 (15.90%)
New medication in 6 months after FC	
Steroids	170 (18.52%)
Immunomodulator	105 (11.44%)
Anti-TNF	47 (5.12%)
Any of these	239 (26.03%)

FC, fecal calprotectin; IQR, interquartile range; TNF, tumor necrosis factor.

Demographic and biomarker data on the cohort stratified by whether the patients reached the composite end point or not are shown in Table 2. On univariable Cox proportional hazards analysis, FC level was associated strongly with an increased risk of reaching the primary end point (Table 3), with a hazard ratio of 1.79 (95% CI, 1.50–2.14; *P* = 1.9 × 10^-10^) for log_10_ (FC). The only other blood tests nominally associated with FC on univariable analysis were C-reactive protein (*P* = .016), hemoglobin (*P* = .011), and platelets (*P* = .003). There also were associations with younger age at diagnosis (*P* = .010), female sex (*P* = .021), prior immunomodulator use (*P* = .012), and symptoms at index visit (*P* = 1.2 × 10^-7^). Smoking status, previous intestinal resection, previous anti–tumor necrosis factor, and time period of FC measurement (before vs after 2008) were not associated with the primary end point, and there was no significant difference in the time since diagnosis at the index FC.

**Table 2:** Demographics and Investigations at Index Visit Stratified by Whether Individuals Reached the Composite Primary End Point of Progression in Montreal Behavior, Surgery, or Hospitalization

Variable		Primary end point		*P*
		Not reached	Reached	
Sex				
Male		235 (42.4%)	105 (32.5%)	.005
Female		320 (57.7%)	217 (67.4%)	
Age at diagnosis, *y*		28.2 (20.9–45.0)	24.7 (17.9–38.1)	2.3 × 10^-4^
Age at calprotectin, *y*		41.9 (30.0–56.3)	38.0 (26.7–49.8)	2.7 × 10^-4^
Months to first calprotectin		69.3 (13.4–183.8)	85.1 (20.0–189.5)	.234
Montreal location				
L1		167 (30.3%)	110 (34.5%)	1.7 × 10^-4^
L2		224 (40.6%)	88 (27.6%)	
L3		159 (28.8%)	117 (36.7%)	
Smoker at visit				
No		263 (75.1%)	142 (68.9%)	.115
Yes		87 (24.9%)	64 (31.1%)	
Previous resection		231 (41.6%)	146 (45.3%)	.289
Previous immunomodulator		255 (45.9%)	166 (51.6%)	.123
Previous anti-TNF		110 (19.8%)	68 (21.1%)	.664
Symptomatic at index visit		195 (53.4%)	162 (78.3%)	2.4 × 10^-9^
Investigation	n			
Fecal calprotectin, *ug/g*	877	180 (50–665)	432 (136–998)	6.9 × 10^-12^
CRP, *mg/L*	375	7 (3–19)	10 (4–27)	.023
ESR, *mm/h*	202	21 (11–36)	26 (14–41)	.045
Albumin, *g/L*	350	40 (36–43)	38 (32–43)	.097
Hemoglobin, *g/L* (scaled to male range)	500	148 (139–155)	145 (133–154)	.009
WCC, ×10^9^/L	507	7.5 (5.9–9.4)	7.3 (5.8–9.5)	.785
Platelets, ×10^9^/L	489	277 (225–342)	305 (249–377)	4.9 × 10^-4^

NOTE. Values shown are medians (interquartile ranges) and numbers (percentages) as appropriate. *P* values were calculated using the Mann–Whitney *U* and Fisher exact tests for continuous and categoric data, respectively.

CRP, C-reactive protein; ESR, erythrocyte sedimentation rate; TNF, tumor necrosis factor; WCC, white cell count.

**Table 3:** Univariable and Multivariable Analyses Using Cox Proportional Hazards Models for Time to Reaching Primary End Point

Variable	Univariable		Multivariable	
	HR (95% CI)	*P*	HR (95% CI)	*P*
Sex, female	1.31 (1.04–1.65)	.021	1.66 (1.23–2.24)	.001
Age at diagnosis, *y*	0.99 (0.98–1.00)	.010		
Age at calprotectin, *y*	0.99 (0.98–1.00)	.001	0.99 (0.98–1.00)	.010
No ileal involvement (Montreal L2)	0.66 (0.51–0.84)	7.9 × 10^-4^	0.60 (0.44–0.82)	.001
Previous immunomodulator	1.32 (1.06–1.64)	.012	1.39 (1.04–1.84)	.024
Previous anti-TNF	1.12 (0.86–1.46)	.411		
Symptomatic at index visit	2.45 (1.76–3.42)	1.2 × 10^-7^	2.07 (1.46–2.93)	4.1 × 10^-5^
Fecal calprotectin, *ug/g*^a^	1.79 (1.50–2.14)	1.9 × 10^-10^	1.49 (1.17–1.89)	.001
CRP, *mg/L*^a^	1.44 (1.07–1.93)	.016		
Hemoglobin, *g/L* (scaled to male range)	0.99 (0.98–1.00)	.011		
Platelets, ×10^9^/L	1.00 (1.00–1.00)	.003		

CRP, C-reactive protein; HR, hazard ratio; TNF, tumor necrosis factor.

a Variable log_10_ transformed prior to use in the model. Hazard ratio is for each 10-fold increase in the variable.

On multivariable Cox proportional hazards analysis, disease progression was associated independently with increased FC level, female sex, younger age, ileal/ileocolonic disease, previous immunomodulator use, and symptoms (Table 3).

A further analysis was performed to explore the effect of changes in treatment before and after measurement of calprotectin (Supplementary Table 1). This was restricted to patients who did not have disease progression and were not censored within the first 6 months. There were no significant associations with changes in medication in the 3 months leading up to the FC measurement. Use of steroids in the 6 months after calprotectin was associated significantly with disease progression (hazard ratio, 1.5; 95% CI, 1.16–2.03; *P* = .003). However, this was no longer significant in a multivariable analysis that also included the FC result (Supplementary Table 2).

**Figure 3:**
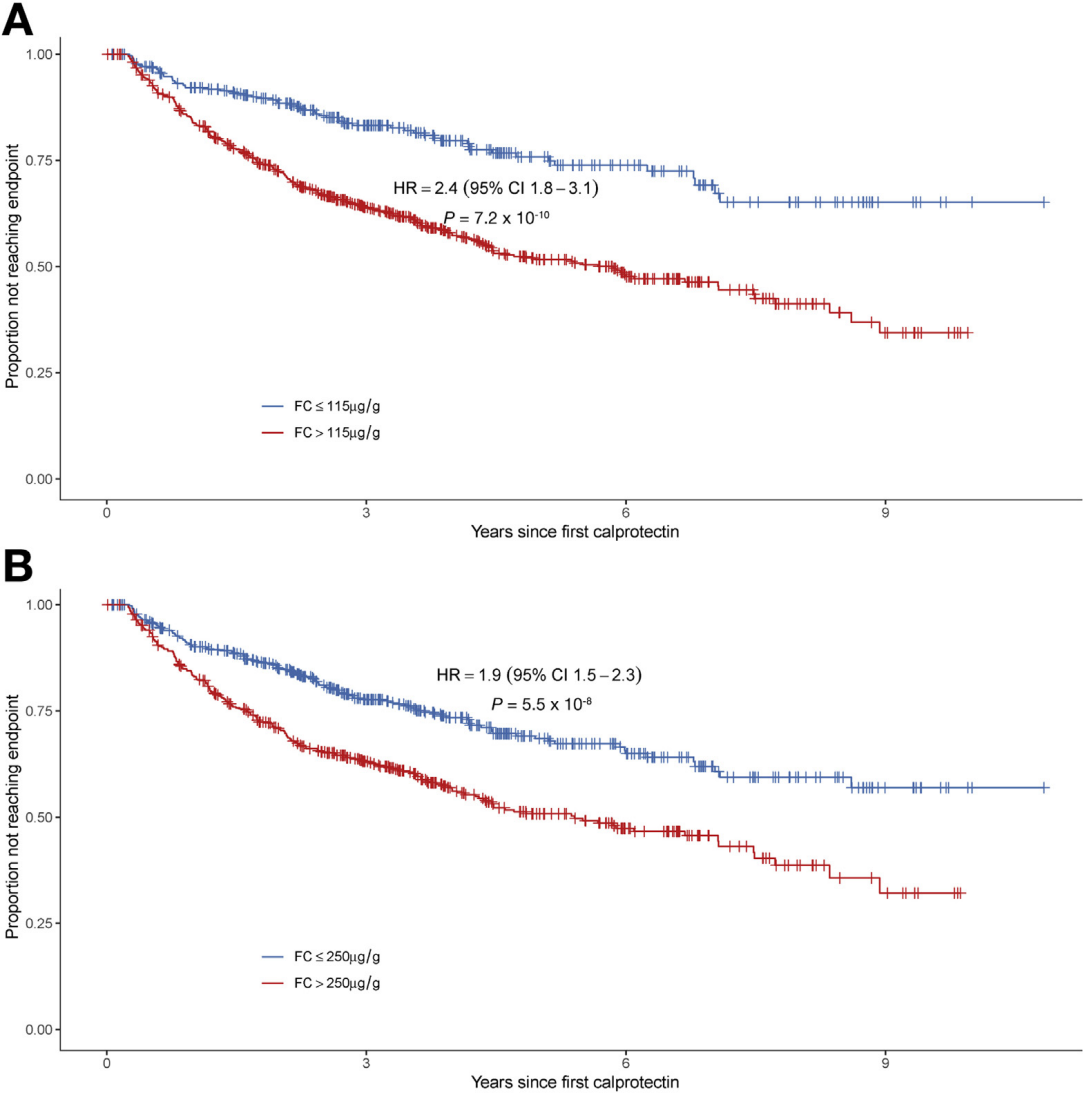
Kaplan–Meier plot of time to reach primary end point stratified by fecal calprotectin (FC) levels (*A*) greater than 250 μg/g and (*B*) greater than 115 μg/g at the index visit. The outcome of maintained digestive health is defined here as the inverse of the primary study end point (a composite of progression in Montreal behavior, hospitalization, or surgery). HR, hazard ratio.

**Supplementary Figure 1:**
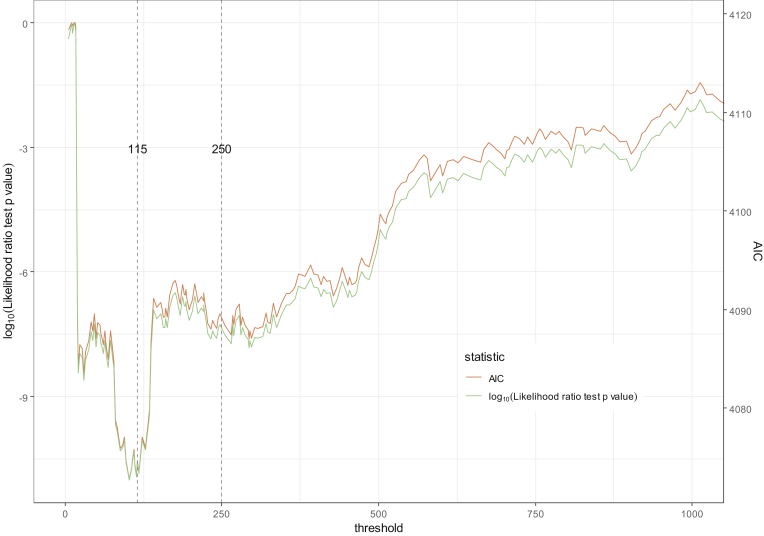
Comparison of Akaike Information Criterion (AIC) and the likelihood ratio test *P* value for Cox proportional hazards models at different thresholds of fecal calprotectin.

**Supplementary Figure 2:**
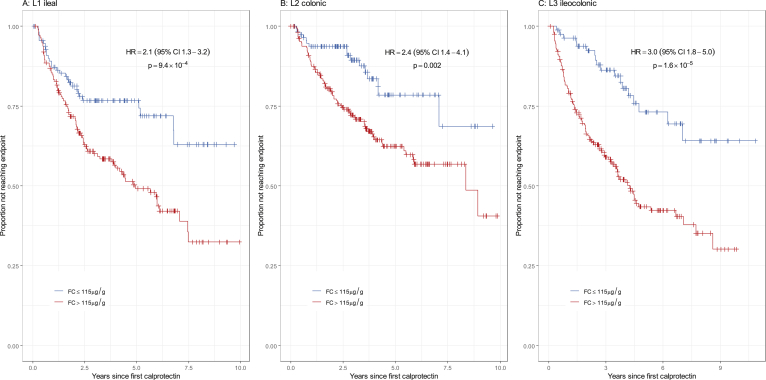
Kaplan–Meier plot of time to reach primary end point stratified by fecal calprotectin (FC) greater than 115 μg/g at the index visit and by Montreal location. The outcome of maintained digestive health is defined here as the inverse of the primary study end point (a composite of progression in Montreal behavior, hospitalization, or surgery). (*A*) L1 ileal. (*B*) L2 colonic. (*C*) L3 ileocolonic. HR, hazard ratio.

**Supplementary Figure 3:**
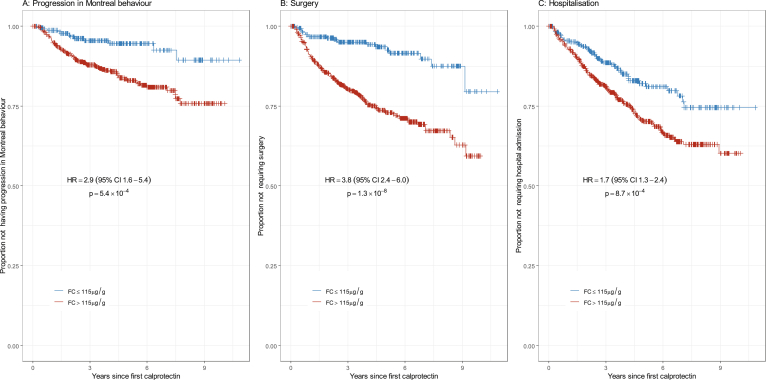
Kaplan–Meier plots of the 3 separate secondary outcome measures stratified by fecal calprotectin (FC) level greater than 115 μg/g. (*A*) Progression in Montreal behavior. (*B*) Surgery. (*C*) Hospitalization. HR, hazard ratio.

**Supplementary Figure 4:**
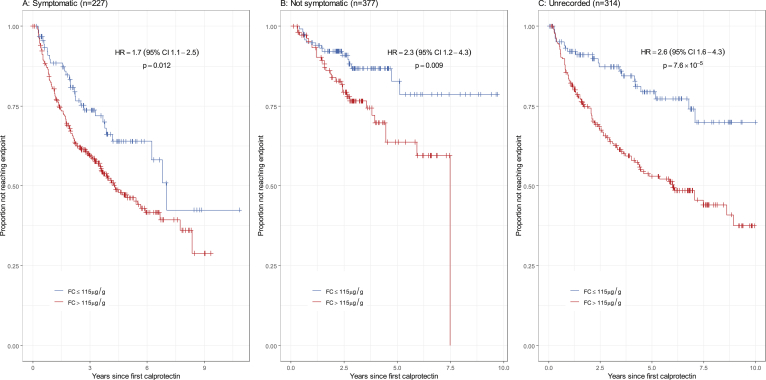
Kaplan–Meier plots of the primary end point stratified by fecal calprotectin (FC) level greater than 115 μg/g and by the presence of symptoms at the index visit. The outcome of maintained digestive health is defined here as the inverse of the primary study end point (a composite of progression in Montreal behavior, hospitalization, or surgery). (*A*) Symptomatic (n = 227). (*B*) Not symptomatic (n = 377). (*C*) Unrecorded (n = 314). HR, hazard ratio.

At a threshold greater than 250 μg/g FC, the hazard ratio for reaching the primary end point was 1.9 (95% CI, 1.5–2.3; *P* = 5.5 × 10^-8^) (Figure 3*A*). By using analyses of different FC thresholds (Supplementary Figure 1), the most significant difference in progression to the primary composite end point was a cut-off value of 115 μg/g (Figure 3*B*), yielding a hazard ratio on multivariable analysis of 2.4 (95% CI, 1.8–3.1; *P* = 7.2 × 10^-10^). Differences in progression were seen in all 3 principal Montreal locations (L1, L2, and L3) (Supplementary Figure 2), in all 3 secondary end points (Supplementary Figure 3), and independent of symptom status at the index visit (Supplementary Figure 4).

Using the Kaplan–Meier estimates, the positive predictive value of an index FC greater than 115 μg/g was 28%, 43%, 52%, and 59% at 2, 4, 6, and 8 years, respectively. The negative predictive value of an index FC of 115 μg/g or less was 88%, 80%, 74%, and 65% at 2, 4, 6, and 8 years, respectively.

**Supplementary Figure 5:**
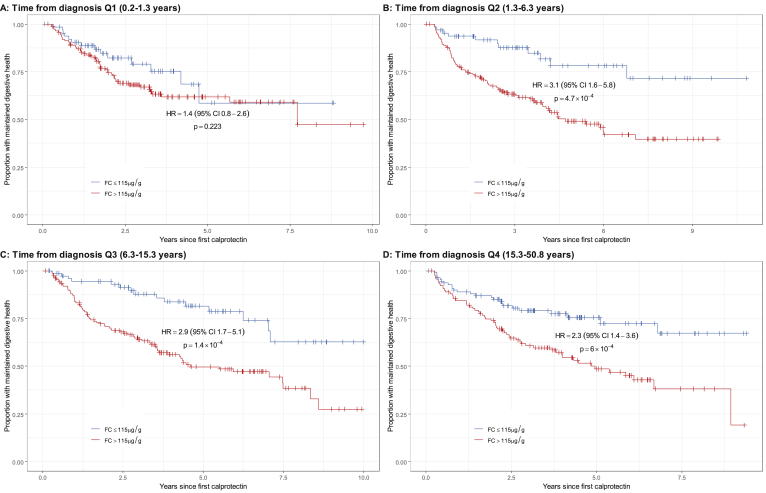
Kaplan–Meier plots of the primary end point stratified by fecal calprotectin (FC) level greater than 115 μg/g and by the quartile of time between diagnosis and first FC measurement. The outcome of maintained digestive health is defined here as the inverse of the primary study end point (a composite of progression in Montreal behavior, hospitalization, or surgery). (*A*) Time from diagnosis Q1 (0.2–1.3 y). (*B*) Time from diagnosis Q2 (1.3–6.3 y). (*C*) Time from diagnosis Q3 (6.3–15.3 y). (*D*) Time from diagnosis Q4 (15.3–50.8 y).

In a sensitivity analysis by quartiles of time from diagnosis to first FC measurement, the association between calprotectin and disease progression was seen for quartiles 2 to 4, but not for the patients in the first quartile; these patients had 3 to 15.5 months between their diagnosis and first FC measurement (Supplementary Figure 5).

**Supplementary Figure 6:**
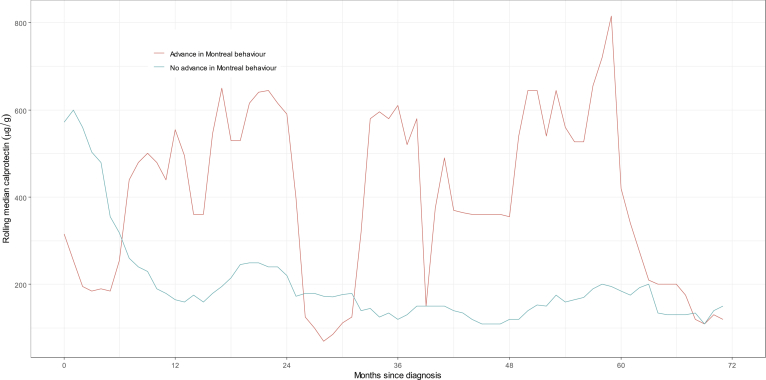
Rolling median fecal calprotectin level by time since diagnosis stratified by subsequent progression in Montreal behavior.

We performed an exploratory analysis using all of the available FC data in CD patients and excluding FC measurement taken when patients had symptoms. This analysis included 1456 FCs from 396 patients. The rolling median FC can be seen clearly to differ between those 35 of 396 patients with a subsequent progression in Montreal behavior and those who did not have a progression (Supplementary Figure 6).

## Discussion

This study shows that increased FC concentration is associated with increased disease progression, both as defined by a composite primary end point of advance in Montreal luminal behavior, surgical resection, and hospitalization, and by each of these end points when considered individually.

Mucosal healing is recognized as a target for therapy in Crohn’s disease, with poorer prognosis and a higher risk of surgery associated with increased endoscopic disease activity.^4^, ^5^, ^6^, ^7^, ^8^ There is a strong correlation between FC, endoscopic disease activity, and ulcer depth.^12^, ^28^ Our data show more directly that increased FC can be used as a marker of increased risk of progression.

Although absolute index FC levels were lower in L1 patients, FC concentration better predicted poorer outcomes in patients with L1/L3 rather than L2 disease distribution. Patients with active colonic disease may be more likely to show symptoms, and thus have an earlier intervention. In contrast, patients with active ileal disease may tolerate a higher level of subclinical inflammation, resulting in a delay of treatment with a greater risk of progression and complications.

Other variables associated with an adverse outcome in our analysis included younger age, which has been identified previously as an adverse prognostic factor,^1^ and previous immunomodulator use, which is likely to be a marker for a more aggressive prior disease course. Symptomatically active disease was associated with an increased rate of disease progression independently of increased FC. This validates a treat-to-target approach aiming for a combination of resolution of symptoms as well as mucosal healing, with FC being a marker of the latter.

Thresholds for prediction of disease relapse have varied across the literature, influenced by the disease cohort being studied and the assay used. Several studies have identified a cut-off value of 250 μg/g as being useful to distinguish active from inactive disease.^20^, ^22^, ^29^ In the present study, the optimal separation between survival curves for progression of disease was seen using a lower threshold of 115 μg/g, suggesting that lower levels of inflammatory activity still may be associated with an adverse outcome. However, any such threshold needs to be interpreted in the context of the methods of FC extraction and measurement. For example, others have shown significant variability in FC measurement between weight-based and other methods of FC extraction, and similarly when comparing enzyme-linked immunosorbent assay kits from different manufacturers.^30^, ^31^

We have shown that increased FC concentration at any point in the disease course beyond the first year correlates with poorer outcome. Previous studies have shown an increase in symptomatic relapse in patients with an increase of FC level,^20^, ^21^, ^22^ our study further indicates that this is associated with an increase in disease progression. The CALM study recently showed better outcomes at 52 weeks when a strategy incorporating symptoms, C-reactive protein level, and FC level was compared with clinical disease activity alone.^32^ Together, these data now clearly support a treat-to-target strategy combining a patient-reported symptom score with FC as a marker of mucosal inflammation.

Strengths of the present study include the large number of patients and duration of follow-up evaluation, with a median follow-up time after index FC of longer than 4 years. A clinically relevant definition of disease progression was selected a priori, and rich phenotype information was available. Restricting measurement of end points to at least 90 days after the index FC measurement should reduce bias from measuring disease activity associated with an exacerbation that went on to cause hospital admission or surgical resection. It also can be observed that the survival curves in Figure 3 and Supplementary Figure 3, Supplementary Figure 4, Supplementary Figure 5 continue to separate for many months after the index FC. This suggests that identification of mucosal inflammation at any point in patient follow-up evaluation, even at relatively modest levels previously considered acceptable (ie, FC, 115–250 ug/g), should warrant careful monitoring and a low threshold for treatment escalation decisions.

Limitations of this study relate to its retrospective nature. FC measurements were not collected at fixed intervals, but as determined by the treating clinician. However, routine monitoring of FC levels including in asymptomatic patients was established quite early on in Edinburgh after the full roll-out of the FC test in 2005. The study also was performed at a single center, which may reduce heterogeneity, but at the expense of generalizability. Nonetheless, although the Western General Hospital is a referral center, it also has a large secondary care population from the local catchment. Finally, medication data were completed as accurately as was possible, but it is possible some courses of steroids, particularly those in primary care, may have been missed. This is unlikely to have introduced any systematic bias.

In conclusion, we have shown in this study that increased FC levels are associated with an increased risk of disease progression over time in CD. Further studies should continue to explore the utility of repeated FC measurements, and to assess whether intervention based on FC concentration can alter disease outcome.

## Figures and Tables

**Supplementary Table 1:** Univariable Survival Analysis of the Primary Composite End Point Stratified by FC and Changes of Medication 3 Months Before and 6 Months After Calprotectin

Variable	HR (95% CI)	*P*
Sex, female	1.30 (1.01–1.66)	.042
Age at diagnosis, *y*	0.99 (0.98–1.00)	.017
Age at calprotectin, *y*	0.99 (0.98–1.00)	.001
Montreal location L2 vs L1/L3	0.66 (0.50–0.86)	.002
Smoker	1.13 (0.82–1.55)	.467
Previous resection	0.97 (0.77–1.23)	.796
Previous immunomodulatory	1.29 (1.02–1.63)	.036
Previous anti-TNF	1.13 (0.82–1.57)	.450
Symptoms or HBI	2.25 (1.58–3.20)	6.4 × 10^-6^
FC level >115 μg/g	2.36 (1.76–3.17)	1.1 × 10^-8^
FC, log_10_	1.80 (1.49–2.19)	2.6 × 10^-9^
New medication in 3 months before FC		
Steroids	0.84 (0.55–1.29)	.436
Immunomodulator	1.22 (0.75–1.99)	.429
Anti-TNF	1.76 (0.72–4.26)	.213
Any of these	1.10 (0.79–1.53)	.567
The outcome of maintained digestive health		
Steroids	1.53 (1.16–2.03)	.003
Immunomodulator	1.26 (0.88–1.80)	.211
Anti-TNF	1.25 (0.73–2.13)	.424
Any of these	1.48 (1.15–1.91)	.002

NOTE. Patients reaching the end point or censored in the first 6 months were excluded.

FC, fecal calprotectin; HBI, Harvey Bradshaw Index; HR, hazard ratio; TNF, tumor necrosis factor.

**Supplementary Table 2:** Multivariable Cox Proportional Hazards Model of the Association Between Primary Composite End Point, FC, and Changes in Medication Before and After the Calprotectin

Term	HR (95% CI)	*P*
FC, log_10_	1.78 (1.45–2.19)	5.0 × 10^-8^
Anti-TNF within 3 months before	1.45 (0.58–3.61)	.43
Immunomodulators within 3 months before	1.15 (0.69–1.91)	.60
Steroids within 3 months before	0.70 (0.45–1.08)	.11
Anti-TNF within 6 months after	0.89 (0.51–1.55)	.69
Immunomodulators within 6 months after	0.99 (0.67–1.45)	.95
Steroids within 6 months after	1.20 (0.88–1.62)	.25

FC, fecal calprotectin; HR, hazard ratio; TNF, tumor necrosis factor.
